# Draft Genome Sequence of Leifsonia poae Strain BS71, Isolated from a Drought Microcosm

**DOI:** 10.1128/mra.00951-21

**Published:** 2022-01-06

**Authors:** Hanaa Ahmed, Kristen M. DeAngelis, Maureen A. Morrow

**Affiliations:** a Department of Biology, State University of New York at New Paltz, New Paltz, New York, USA; b Department of Microbiology, University of Massachusetts, Amherst, Massachusetts, USA; University of Maryland School of Medicine

## Abstract

We report the draft genome sequence of Leifsonia poae strain BS71. This bacterium was isolated from a low soil moisture content model soil microcosm inoculated with forest soil that had been subject to chronic warming.

## ANNOUNCEMENT

Climate change is expected to increase drought conditions globally (1). To examine climate effects on soils, a field warming experiment was established in the Harvard Research Forest (HRF), a temperate forest ecosystem in Petersham, MA (42.54°N, −72.18°W) (2). In October 2017, mineral horizon soil was collected from heated plots. A microcosm was generated by inoculating a 0.8-μm filtered soil slurry into an artificial soil microcosm at 30% water content and incubating it for 4 months at 15°C, feeding it weekly with cellobiose and NH_4_NO_3_, as described previously (3). These conditions were hypothesized to enrich for bacteria capable of growing in low soil moisture. Strain BS71 was isolated from the microcosm on a 1% glucose/0.4% potato infusion (Sigma-Aldrich, St. Louis, MO) agar plate, pH 6, under aerobic conditions for 8 days at 25°C in the dark. BS71 was identified as Leifsonia poae by analyzing the 16S rRNA PCR product produced with the 27F/1492R primer pair (4) using IDTAXA (5).

BS71 DNA was prepared for sequencing by growing a single colony on 10% tryptic soy agar at 25°C for 7 days in the dark, scraping the biomass, and extracting the DNA using the Qiagen genomic DNA protocol (Valencia, CA). Whole-genome sequencing was completed at the University of Massachusetts Medical School (UMMS) sequencing center. The DNA was sheared using a Bioruptor device (Diagenode, NJ) to a mean size of 20 kb. A PacBio SMRTbell library kit was used to construct a library, which was sequenced on the PacBio RS II platform. The 150,292 raw reads, generated from a single cell, were filtered using the SMRT portal P-filter module (minimum subread length, 50 nucleotides; minimum polymerase quality, 75; and minimum polymerase read length, 50 nucleotides), and the resultant 69,059 filtered reads had a read *N*_50_ value of 7,302 bases.

The genome was assembled using sprai v0.9.9.23 (https://anaconda.org/bioconda/sprai) and Canu v1.5 (6). The final draft assembly contained 5 contigs (contig *N*_50_, 3.98 Mb) and was estimated to be 98.99% complete and 0.063% contaminated using CheckM v1.0.18 (7) in KBase (8). Gene annotations were completed within JGI’s Integrated Microbial Genomes (IMG) MGAP v4.16.5 (9) with the gene calling program Prodigal v2.6.3 (10, 11). Default parameters were used for all software except where noted. The genome is 4,144,138 bp (coverage, 94.3×), with a GC content of 67.84%, and is predicted to encode 3,961 proteins, a single rRNA operon, and 45 tRNA genes.

A manually curated list of drought-associated genes was compared between BS71 and the 20 *Leifsonia* genomes with the greatest 16S rRNA gene homology within IMG’s database. A greater number of beta-glucosidase (EC 3.2.1.21) annotated genes were present in BS71 (24 genes), compared to 10 or fewer such annotated genes in the other *Leifsonia* (Table 1). Beta-glucosidase enzymes are diverse and play an important role in biomass conversion of recalcitrant carbon (12). The presumptive BS71 drought tolerance is also supported by the annotation of two distinct aquaporin genes and genes for the production and transport of osmoprotectants. This genome supports the hypothesis that the drought conditions characteristic of climate change may select for bacteria with drought-associated traits.

**TABLE 1:** Protein-protein BLAST results for beta-glucosidase (EC 3.2.1.21) annotated genes

GeneID	Taxon identified by BLASTp highest score	Identity (%)	No. of amino acids	IMG annotation description
2806535936	*Humibacter* sp. strain WJ7-1	71	846	Glycoside hydrolase family 3 C-terminal domain-containing protein
2806536034	Streptacidiphilus fuscans	63	401	Family 1 glycosyl hydrolase
2806536039	Leifsonia shinshuensis	70	96	Family 1 glycosyl hydrolase
2806536324	Microbacterium azadirachtae	60	788	Glycoside hydrolase family 3 C-terminal domain-containing protein
2806536495	Leifsonia shinshuensis	84	599	Glycoside hydrolase family 3 protein
2806536706	*Leifsonia* sp. strain NCR5	81	831	Glycoside hydrolase family 3 C-terminal domain-containing protein
2806536737	*Frondihabitans* sp. strain 762G35	73	776	Glycoside hydrolase family 3 C-terminal domain-containing protein
2806536747	*Leifsonia* sp. strain Root227	94	391	Family 1 glycosyl hydrolase
2806536801	*Leifsonia* sp. strain NCR5	82	617	Glycoside hydrolase family 3 C-terminal domain-containing protein
2806537199	*Leifsonia* sp. strain PS1209	79	501	Beta-glucosidase
2806537254	Plantibacter flavus	65	592	Glycoside hydrolase family 3 C-terminal domain-containing protein
2806537278	*Microbacterium* sp. strain Root61	91	761	Glycoside hydrolase family 3 C-terminal domain-containing protein
2806538045	*Rathayibacter* sp. strain AY1A3	67	805	Glycoside hydrolase family 3 C-terminal domain-containing protein
2806538050	*Nonomuraea* sp. strain 160415	61	403	Family 1 glycosyl hydrolase
2806538065	Thermocatellispora tengchongensis	70	746	Glycoside hydrolase family 3 C-terminal domain-containing protein
2806538066	Microbacteriaceae bacterium	78	389	Glycosyl hydrolase family protein
2806538078	Thermocatellispora tengchongensis	64	797	Beta-glucosidase
2806538079	Unclassified *Leifsonia*	77	609	MULTISPECIES: glycoside hydrolase family 3 C-terminal domain-containing protein
2806538223	*Plantibacter* sp. strain YR521	69	781	Glycoside hydrolase family 3 C-terminal domain-containing protein
2806535250	*Actinoplanes* sp. strain OR16	68	578	Glycoside hydrolase family 3 C-terminal domain-containing protein
2806535263	*Streptomyces* sp. strain yr375	83	409	Family 1 glycosyl hydrolase
2806535267	*Leifsonia* sp. Root227	88	751	ABC transporter substrate-binding protein
2806535268	*Leifsonia* sp. Root227	86	786	Glycoside hydrolase family 3 C-terminal domain-containing protein
2806534677	Mycobacterium sp.	63	747	Glycoside hydrolase family 3 C-terminal domain-containing protein

### Data availability.

The 16S PCR product sequence accession number is OL515151. The raw whole-genome sequence reads are available in GenBank under the BioProject accession number PRJNA745001. The Sequence Read Archive (SRA) accession number is SRR15142240, and the nucleotide sequence accession number is JAIHLP000000000. The annotation reported in this study is available at the Joint Genome Institute as the Leifsonia poae BS71 first assembly (https://img.jgi.doe.gov/cgi-bin/m/main.cgi?section=TaxonDetail&page=taxonDetail&taxon_oid=2806310494).

## References

[1] Pokhrel Y, Felfelani F, Satoh Y, Boulange J, Burek P, Gädeke A, Gerten D, Gosling SN, Grillakis M, Gudmundsson L, Hanasaki N, Kim H, Koutroulis A, Liu J, Papadimitriou L, Schewe J, Müller Schmied H, Stacke T, Telteu C-E, Thiery W, Veldkamp T, Zhao F, Wada Y. 2021. Global terrestrial water storage and drought severity under climate change. Nat Clim Chang 11:226–233. doi:10.1038/s41558-020-00972-w.

[2] Melillo JM, Frey SD, DeAngelis KM, Werner WJ, Bernard MJ, Bowles FP, Pold G, Knorr MA, Grandy AS. 2017. Long-term pattern and magnitude of soil carbon feedback to the climate system in a warming world. Science 358:101–105. doi:10.1126/science.aan2874. 28983050

[3] Domeignoz-Horta LA, Pold G, Liu X-JA, Frey SD, Melillo JM, DeAngelis KM. 2020. Microbial diversity drives carbon use efficiency in a model soil. Nat Commun 11:3684. doi:10.1038/s41467-020-17502-z. 32703952 PMC7378083

[4] Lane DJ. 1991. 16S/23S rRNA Sequencing, p 115–175. *In* Stackebrandt E, Goodfellow M (ed), Nucleic acid techniques in bacterial systematics. John Wiley and Sons, New York, NY.

[5] Murali A, Bhargava A, Wright ES. 2018. IDTAXA: a novel approach for accurate taxonomic classification of microbiome sequences. Microbiome 6:140. doi:10.1186/s40168-018-0521-5. 30092815 PMC6085705

[6] Koren S, Walenz BP, Berlin K, Miller JR, Bergman NH, Phillippy AM. 2017. Canu: scalable and accurate long-read assembly via adaptive k-mer weighting and repeat separation. Genome Res 27:722–736. doi:10.1101/gr.215087.116. 28298431 PMC5411767

[7] Parks DH, Imelfort M, Skennerton CT, Hugenholtz P, Tyson GW. 2015. CheckM: assessing the quality of microbial genomes recovered from isolates, single cells, and metagenomes. Genome Res 25:1043–1055. doi:10.1101/gr.186072.114. 25977477 PMC4484387

[8] Arkin AP, Cottingham RW, Henry CS, Harris NL, Stevens RL, Maslov S, Dehal P, Ware D, Perez F, Canon S, Sneddon MW, Henderson ML, Riehl WJ, Murphy-Olson D, Chan SY, Kamimura RT, Kumari S, Drake MM, Brettin TS, Glass EM, Chivian D, Gunter D, Weston DJ, Allen BH, Baumohl J, Best AA, Bowen B, Brenner SE, Bun CC, Chandonia J-M, Chia J-M, Colasanti R, Conrad N, Davis JJ, Davison BH, DeJongh M, Devoid S, Dietrich E, Dubchak I, Edirisinghe JN, Fang G, Faria JP, Frybarger PM, Gerlach W, Gerstein M, Greiner A, Gurtowski J, Haun HL, He F, Jain R, Joachimiak MP, Keegan KP, Kondo S, et al. 2018. KBase: the United States Department of Energy Systems Biology Knowledgebase. Nat Biotechnol 36:566–569. doi:10.1038/nbt.4163. 29979655 PMC6870991

[9] Huntemann M, Ivanova NN, Mavromatis K, Tripp HJ, Paez-Espino D, Palaniappan K, Szeto E, Pillay M, Chen I-MA, Pati A, Nielsen T, Markowitz VM, Kyrpides NC. 2015. The standard operating procedure of the DOE-JGI Microbial Genome Annotation Pipeline (MGAP v.4). Stand Genomic Sci 10:86. doi:10.1186/s40793-015-0077-y. 26512311 PMC4623924

[10] Chen I-MA, Chu K, Palaniappan K, Pillay M, Ratner A, Huang J, Huntemann M, Varghese N, White JR, Seshadri R, Smirnova T, Kirton E, Jungbluth SP, Woyke T, Eloe-Fadrosh EA, Ivanova NN, Kyrpides NC. 2019. IMG/M v.5.0: an integrated data management and comparative analysis system for microbial genomes and microbiomes. Nucleic Acids Res 47:D666–D677. doi:10.1093/nar/gky901. 30289528 PMC6323987

[11] Hyatt D, Chen G-L, LoCascio PF, Land ML, Larimer FW, Hauser LJ. 2010. Prodigal: prokaryotic gene recognition and translation initiation site identification. BMC Bioinformatics 11:119. doi:10.1186/1471-2105-11-119. 20211023 PMC2848648

[12] Ketudat Cairns JR, Esen A. 2010. β-Glucosidases. Cell Mol Life Sci 67:3389–3405. doi:10.1007/s00018-010-0399-2. 20490603 PMC11115901

